# A Multi-Purpose Shallow Convolutional Neural Network for Chart Images

**DOI:** 10.3390/s22207695

**Published:** 2022-10-11

**Authors:** Filip Bajić, Ognjen Orel, Marija Habijan

**Affiliations:** 1 University Computing Centre, University of Zagreb, 10000 Zagreb, Croatia; 2 Faculty of Electrical Engineering, Computer Science and Information Technology Osijek, 31000 Osijek, Croatia

**Keywords:** chart classification, data visualization, convolutional neural network, Siamese neural network, shallow neural network, generative adversarial network

## Abstract

Charts are often used for the graphical representation of tabular data. Due to their vast expansion in various fields, it is necessary to develop computer algorithms that can easily retrieve and process information from chart images in a helpful way. Convolutional neural networks (CNNs) have succeeded in various image processing and classification tasks. Nevertheless, the success of training neural networks in terms of result accuracy and computational requirements requires careful construction of the network layers’ and networks’ parameters. We propose a novel Shallow Convolutional Neural Network (SCNN) architecture for chart-type classification and image generation. We validate the proposed novel network by using it in three different models. The first use case is a traditional SCNN classifier where the model achieves average classification accuracy of 97.14%. The second use case consists of two previously introduced SCNN-based models in parallel, with the same configuration, shared weights, and parameters mirrored and updated in both models. The model achieves average classification accuracy of 100%. The third proposed use case consists of two distinct models, a generator and a discriminator, which are both trained simultaneously using an adversarial process. The generated chart images are plausible to the originals. Extensive experimental analysis end evaluation is provided for the classification task of seven chart classes. The results show that the proposed SCNN is a powerful tool for chart image classification and generation, comparable with Deep Convolutional Neural Networks (DCNNs) but with higher efficiency, reduced computational time, and space complexity.

## 1. Introduction

A “Chart” is well known as a graphical representation of tabular data. Charts help people understand large (often numeric) quantities of data, show the relation between the data, point out important information, and help present that information to others who may or may not have prior knowledge of it. The data in charts are represented by graphical symbols (bars, lines, circles) that have been used since the 18th century [1,2]. Since then, charts have expanded to every aspect of life, from mathematics and statistics to engineering, chemistry, and medicine, and are common in business documents, media, and scientific publications [3]. Understanding these graphical representations (images) is a well-studied challenge affecting blind or visually impaired people and machines. While humans can easily decode chart images, machines cannot [4]. Due to the challenges mentioned above, various scientific research has been conducted to increase the availability of chart images to enable a more efficient chart type classification and more accurate and detailed interpretation of the content. The first step in successfully retrieving information from a chart image is to classify that image into one of many existing chart-type classes. After the successful completion of the first step, the chart image can be further processed with data extraction algorithms, optical character recognition systems, or systems that generate a description of the chart image using natural language processing algorithms.

In recent years, Artificial Neural Networks (ANNs) have been used in many classification tasks to solve complex computer vision challenges. The Convolutional Neural Network (CNN), a subclass of ANNs, is the most successful and widely used neural network that achieves state-of-the-art results in many pattern recognition tasks [5]. Although high accuracy, scalability, and flexibility are advantages of CNNs, there are also a few disadvantages that have a huge impact on expected results. The main disadvantage is choosing the correct CNN architecture, which affects the required dataset size, computational power, and time to train the network. Some of the most used CNN architectures for chart type classification are LeNet [6], AlexNet [7], VGG [8], GoogLeNet [9], and ResNet [10,11].

The network architecture depends on the number and type of used layers. In the listed architectures, the number of layers ranges from dozens to hundreds and the number of parameters from thousands to millions. The deeper the architecture, the more resources are required, but often better results can be achieved [12]. Although these Deep Convolutional Neural Networks (DCNNs) architectures achieve state-of-the-art results, training them requires vast computational and dataset resources. To train these networks, advanced hardware is required, which is often inaccessible to everyone.

Compared to DCNN, the training of SCNN can be done in less time, and the required computational power and dataset size are significantly reduced. On the other hand, SCNN consists only of a few layers, and the number of parameters is reduced considerably. Due to their simplicity, SCNNs are often overlooked and underestimated, while DCNNs are being researched more, and becoming more advanced.

### Research Contributions

The main novelty presented in this paper is a novel SCNN architecture for chart type classification and image generation. The proposed network consists only of four convolutional layers, two max-pooling layers, and one fully-connected layer. Therefore, it is much more computationally efficient than DCNNs with similar performances. To demonstrate the abilities of this architecture and prove its state-of-the-art, it is implemented in three different use cases.

First, we use proposed SCNN as a traditional (classic) CNN. The achieved average classification accuracy is calculated on seven chart types, and the results are compared against our previous work [11]. The network’s performance is also compared against other publications that achieve state-of-the-art results. The same datasets are used in both networks when comparing the achieved results.

Second, we use proposed SCNN as a Siamese CNN. The Siamese CNN is a network architecture built using two identical networks with the same parameters, configuration, and shared weights. The classification accuracy is calculated on seven chart types, and the results are compared with our previous work [11] and a traditional SCNN. The same datasets are used in all networks when comparing the achieved results. To our knowledge, no other authors report using Siamese CNN architecture in chart-type classification.

Third, proposed SCNN (with minimal modifications) is used as a Generator network and a Discriminator network. The Generative Adversarial Network (GAN) is used to generate new examples of chart images that plausibly could have been drawn from the original dataset. To the best of our knowledge, this work is the first to present findings on using GAN for generating chart images.

## 2. Related Work

In this section, the results of extensive literature research are presented. The section is divided into chart-type classification, SCNNs, and GANs. Each subsection presents the latest publications and highlights the most relevant ones.

### 2.1. Chart-Type Classification

Chart-type classification is a well-studied process in the field of chart understanding. It refers to classifying a chart image, according to the visual content, into one of many chart classes. It is also the most critical part of any fully automated system to retrieve underlying chart information. The earliest publications that deal with chart type classification were written in 2000 by Zhou and Tan [13,14]. In these publications, the authors introduced one of the most used processes for chart-type classification that any system can adopt. The core of the process is to separate textual and graphical content, resulting in two images that are processed independently. After the processing, the results are joined for final classification.

Over the years, authors have used different methods for obtaining chart-type information. The methods can be grouped into four categories: custom algorithm, the model-based approach, Support Vector Machines (SVMs), and ANNs.

Custom algorithm refers to analyzing and extracting features of a chart image on a pixel level and a graphical symbol level. The method is used in conjunction with advanced image preprocessing, consisting of text and graphics separation, edge detection, vectorization, and binarization. Due to the complex image preprocessing, supported chart classes are usually limited to bar, pie, and line charts. The most notable publications that classify chart images based on a pixel level and graphical symbol level are image and graphic reader [15], View [16], Beagle [17], and ChartFuse [18]. In Beagle [17], the authors report average classification accuracy of 86%. In ChartFuse [18], the authors compare different methods that work on a pixel level and propose a method that differentiates colors, textures, structural layout, and illumination details. The proposed method achieves average classification accuracy of 95–97%.

The model-based approach requires a model for each chart class. Each chart class consists of textual and graphical objects that are part of the chart image. The chart image will not be classified if some of these objects are misplaced or left out. The model needs to be specifically adapted for classifying within-class (e.g., bar chart—vertical bar chart). The model-based approach is best described by Mishchenko and Vassilieva [19,20], and the authors report average classification accuracy of 92%.

The SVMs require extracted image features for a classification task. The extracted features are outputted to finite-dimensional space, and a hyperplane is drawn. The hyperplane separates the data into different classes. This hyperplane is also called a decision boundary, as everything that falls to one side will classify as one class, and everything that falls to the other will be considered the other class. The SVMs can work with small datasets; however, setting the hyperplane is challenging when classes share extracted features. The SVMs are best described in View [16], Reverse-Engineering Visualizations [4], and ReVision [21], by Nair et al. [22], and by Shi et al. [23]. ReVision [21], published in 2011 is the most cited publication in the field of chart understanding as it is a system that classifies chart images, extracts underlying data, and redesigns charts. Authors report average classification accuracy of 82%. When SVMs are paired with CNNs (where CNN is a feature extractor), the average classification accuracy goes up to 90%. The conjunction of CNNs and SVMs is best described in VizByWiki [24], Visualizing for the Non-Visual [25], DocFigure [26], and by Kaur and Kiesel [27].

The listed methods achieve competitive results, but CNN’s achieve state-of-the-art results. Chagas et al. [28] compared listed methods and showed that CNNs outperform by roughly 20%. Publications by Bajić and Job [11], Kosemen and Birant [29], Ishihara et al. [30], and Dadhich et al. [31] use custom CNN architectures for chart-type classification. CNNs can also be used out-of-the-box; some are available as pre-trained models. Pre-trained models are trained on large datasets (e.g., ImageNet [32]), and to use them, only the final layers need to be changed and retrained as in Reverse-Engineering Visualizations [4], FigureSeer [33], Chart-Text [34], VizByWiki [24], Visualizing for the Non-Visual [25], DocFigure [26], by Huang [35] and by Arujo et al. [36]. Common to both architectures is the average classification accuracy, in the range from 90% to 100%. The comparison of different CNN architectures is available in Chart decoder [37], in a publication written by Thiyam et al. [38], and in the results of a competition in chart-type classification [39]. The publications show that all architectures perform similarly, with up to 5% divergence.

Used CNN architectures achieve great success in chart-type classification, and all of them can be considered DCNN. Since chart-type classification reached almost perfect scores using DCNNs, authors should redirect their research to SCNNs as SCNNs trained models can be used on personal computers and mobile devices. To our knowledge, no other authors report on using SCNNs for chart-type classification, and this work is the first to present the results in this field.

### 2.2. Shallow Convolutional Neural Networks

The literature does not strictly define an SCNN or how many layers it should consist of. In theory, the perfect SCNN consists only of an input layer, one hidden layer, and an output layer, which is hardly achievable with complex datasets. A broader definition of SCNN would be a network with a minimal number of hidden layers that completes a specific task. When considering the depth of the network, only weight layers are counted. The weight layers are convolutional and fully-connected, as they contain only the parameters that can be learned. When designed properly, these networks are proven to be as effective as DCNNs, which is best described in the following publications.

Li et al. created an SCNN for apple classification [40]. A proposed network consists of seven convolutional layers, three max-pooling layers, and two fully-connected layers. The input image has three channels; its dimensions are 224×224 px. A softmax layer is used for final classification at the end of a network. The results show that the proposed SCNN architecture achieves higher classification accuracy than the SVM or out-of-the-box DCNNs.

Lei et al. created an SCNN to classify images in the fashion-MNIST dataset [12,41]. A novel SCNN employs two convolutional layers, two max-pooling layers, and a fully-connected layer. The input image has one channel; its dimensions are 28×28 px. A softmax layer is used for final classification at the end of a network. The results show that the proposed SCNN architecture performs similarly to DCNN, with up to 3% divergence.

Gorokhovatskyi and Peredrii created multiple SCNNs for pattern recognition tested on different datasets [42]. The networks range from three to five weight layers with different input dimensions. At the end of each network, a sigmoid layer is used for final classification. Authors conclude that SCNNs achieve similar results as DCNNs, but to be successful, SCNNs need to be specially adapted for each dataset.

Wang et al. created an SCNN to detect actinic keratosis [43]. A proposed network consists of two convolutional layers, a max-pooling layer, and a fully-connected layer. The input image has three channels; its dimensions are 64×64 px. A softmax layer is used for final classification at the end of a network. The proposed network is compared with multiple DCNNs. Regarding average classification accuracy, the proposed SCNN outperforms out-of-the-box DCNNs by 10–15%. The SCNNs are also used to detect inferior myocardial infarction [44], acute brain hemorrhage [45], and diabetic retinopathy [46], where they achieve significant improvement in classification performance.

Jain et al. created SCNN for handwritten signature validation [47]. A proposed network has three convolutional layers and one full-connected layer. The input image has one channel; its dimensions are 64×128 px. A softmax layer is used for final classification at the end of a network. The proposed network was tested on multiple datasets and achieved average classification accuracy between 93% and 99%. Golovko et al. [48] created SCNN for handwritten digit classification. The network consists of two convolutional layers, one max-pooling layer, and two fully-connected layers. The input image has one channel; its dimensions are 28×28 px. A softmax layer is used for final classification at the end of a network. The proposed network achieves higher classification accuracy than out-of-the-box DCNN models.

The presented SCNN architectures have a few things in common: small input image size, small kernel size, and a single-digit number of used weight layers. Following these guidelines, in our work, an SCNN for chart type classification is constructed. Other authors also report using SCNNs for image classification with similar results [49,50,51,52].

### 2.3. Generative Adversarial Networks

There are many generators of synthetic chart images on the Web (D3, Vega, Protovis, Plotly, etc.), but all of them require user interaction or parameter definition [53]. The process becomes time-consuming when creating more synthetic chart images. A GAN is used to automate the process of generating synthetic chart images. GANs are frameworks that learn patterns in input data and use that knowledge to generate new data. A successful GAN generates hardly distinguishable data from the original input data.

Goodfellow et al. invented GANs in 2014 [54]. Since then, GANs have been extensively researched, and many different extensions have appeared. Deep Convolutional Generative Adversarial Network (DCGAN), proposed by Radford et al. [55], is a direct extension of the original GAN, which combines GAN and CNN to obtain better training results. Some of the other well-known versions of GANs are Wasserstein Generative Adversarial Network (WGAN) [56], Least Squares Generative Adversarial Network (LSGAN) [57], Disco Generative Adversarial Network (DiscoGAN) [58], and Cycle-Consistent Generative Adversarial Network (CycleGAN) [59]. DCGANs are the most widely used, achieving impressive results in image generation tasks. Evaluating a GAN is challenging, as there is still a lack of objective function that will capture its strengths and limitations. The best qualitative evaluation is manual image inspection. For quantitative evaluation, many methods have been developed, but most authors report Frechet Inception Distance (FID) or Inception Score (IS).

The medical datasets are often limited in quantity and access and protected by ethical/privacy policy [60]. Venu and Ravula used DCGAN to generate X-ray chest images [60]. The generated images are plausible to the originals. These images are later used in training CNN, increasing average classification accuracy. The DCGAN was trained using 1341 images and achieved an FID score is 1.289.

Puttagunta et al. also used DCGAN to generate X-ray chest images [61]. The DCGAN was trained using 934 images and achieved an FID score is 23.78. The authors report that the generated images are close to the original data with manual inspection. Xu et al. [62] used DCGAN to generate voiceprint samples for identifying Parkinson’s disease. The DCGAN was trained using 120 samples and achieved an FID score is 30.18. The authors state that the model can produce diverse high-resolution images. The generated images are used in training CNN where they improve the model’s accuracy and performance. Another example of using DCGAN for medical purposes is BrainGAN [63]. BrainGAN successfully generates brain magnetic resonance images with and without tumors. The DCGAN was trained using 400 images and the results were manually inspected. The natural and generated images were used to train a CNN, achieving better performance than only natural image datasets.

DCGANs are also used in the mechanical and electrical engineering industry. Gao et al. used DCGAN for generating images of small industrial parts (gear face) [64]. DCGAN-generated images were used with traditional data enhancement methods (flip, pan, rotation), which increased the training data size for the CNN. The DCGAN was trained using 50 images and achieved IS score is 3.850. Liu et al. used DCGAN to generate effective fault data of gas turbine signals. The signals were generated in the time/frequency domain. The experiment showed that using generated signals improves the average classification accuracy of fault detection. The DCGAN was trained using 2100 samples, and the results were manually inspected.

In agriculture, DCGANs generated images of defected or diseased plants, leaves, or fruits [65,66,67,68]. In the automotive industry, DCGANs were used to generate traffic signs, participants, and scenes [69,70,71,72].

As presented, GANs contributed to different scientific fields, especially for research with limited datasets. Chart-type classification is also subject to limited datasets, as the designers can use ingenuity when designing a chart image, which often creates new types. To our knowledge, GANs have not yet been used for generating chart images.

### 2.4. Dataset and Implementation Description

The training and evaluation of all proposed methods are done on the Google Collab platform with PyTorch deep learning framework (version 1.12.0), TorchVision extension that includes standard tools for image transformation (version 0.13.0), PyTorch Ignite library for high-level evaluation (version 0.4.9), and enabled Compute Unified Device Architecture acceleration.

This work extends our previous research [11,73] which includes image preprocessing and used datasets. The two convolutional models (SCNN and Siamese SCNN) use a scaled-down image with a preserved aspect ratio and resolution of 64×64 px. Before converting the image to a black-and-white color space, the number of details on the image is reduced. Using different image filters (grayscale, smooth, sharpen, contrast) and pixel recoloring methods, the title, coordinate axes, legend, and any additional elements outside the graphics are removed. Images for GAN are also scaled-down with a preserved aspect ratio but use 64×64 px in red-green-blue color space. From these images, only text elements and legends are removed.

The proposed methods are evaluated on the International Conference on Document Analysis and Recognition (ICDAR) 2019 dataset [3]. One validation set is excluded from the dataset before training the networks. The validation set consists of 20 images per class (140 images in total). This set is never used in any training process, and its images never change. The set consists of seven chart classes: pie (includes pie and donut), line, scatter, vertical box, horizontal box, vertical bar (includes grouped and stacked), and horizontal bar (includes grouped and stacked).

## 3. Proposed Chart Image SCNN Architecture

When DCNNs are introduced to a small dataset, they tend to overfit. Overfitted models learned the training data too well, resulting in low training loss and low generalization ability, i.e., when these models are introduced to the validation set, they perform poorly. Moreover, with increasing networks’ depth a common obstacle is the appearance of vanishing or exploding gradients. As more layers are added to neural network architecture, the loss function (sigmoid) gradients approach zero. This causes the neural network to learn at a slower pace or to stop learning, ultimately accumulating large errors or vanishing by the time information about the gradient reaches the end of the network.

To deal with DCNN computational requirements and solve the challenges mentioned above, we propose a novel SCNN with fewer layers than DCNN and small convolutional kernel size. This SCNN is used and tested in several use cases later on.

**Figure 1:**
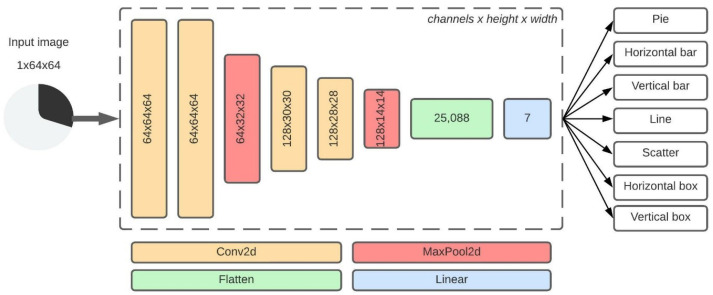
Architecture structure for the proposed SCNN model. The model has five weight layers.

The proposed neural network architecture is shown in Figure 1. The network consists of four convolutional layers, two max-pooling layers, and one fully-connected layer. Due to a fully-connected layer, input data is fixed to 64×64 px. Images are preprocessed and converted to a single channel. Then, two blocks of two convolutional layers are stacked with a max-pooling layer at the end of each block. The convolutional layers are the essential layers of the proposed architecture as they extract the image features. The core idea of Simonyan and Zisserman [8] in their VGG architecture was to use multiple convolutional layers before the max-pooling layer. Authors proved that using double 3×3 receptive field filters has the same effect as using a single 5×5 receptive field filter. The double 3×3 receptive field filter reduces the number of weights but increases the number of layers in the network. A reduced number of weights reduces the computational power, and the increased depth helps the network learn more complex non-linear features. The 3×3 receptive field filter is also the smallest filter that can capture the information from neighboring pixels. Even sized receptive field filters (e.g., 2×2 or 4×4) are not considered as distortion occurs across the layers. Therefore, all convolutional layers in the proposed architecture use a 3×3 receptive field filter. In the first convolutional block, the convolutional stride is fixed to 1 px, and the spatial resolution is preserved with the spatial padding of 1 px (the height and width are preserved). The feature maps obtained by the convolution layers are location-dependent, meaning that the network learns the actual locations of image features in the training process. This occurrence results in reduced network performance. Considering chart images, the location of image features is unimportant, and max-pooling is used after the convolutional block.

Max-pooling selects the maximum pixel value in the receptive field covered by the filter. The first max-pooling layer reduces image features through down-sampling. The layer uses a 2×2 receptive field filter with a stride fixed to 2 px. The size of the max-pooling filter is smaller than the convolutional filter which reduces the size of extracted features by a factor of 2 (the height and width are halved, but the size of a channel is preserved). Reducing the dimensions of extracted features reduces the number of weights in the network. The second convolutional block is similar to the first, except the spatial resolution is not preserved as spatial padding of 0 px is used. The edges of the image do not contain meaningful data (only white space), and the dimensions can be reduced, which reduces the number of weights in the network. At the end of the block, the second max-pooling layer is used, with the same parameters as the first max-pooling layer. The output of the max-pooling layers is connected to the input of the fully-connected layer.

A fully-connected layer connects every input to every output neuron and flattens the input to a single vector of size 25,088 × 1 × 1. The 25,088 is the number of neurons calculated by multiplying the number of channels, height, and width from the previous layer (128 × 14 × 14 = 25,088). A softmax operation is used for the final classification at the end of the proposed architecture. Softmax transforms input values to range 0–1 which are then interpreted as probabilities. The model outputs probabilities for seven chart classes.

### 3.1. Chart Image SCNN Use-Cases and Evaluation

The proposed network is used as a model in three cases: a traditional SCNN, a Siamese SCNN, and minimal modifications as a Generator and Discriminator in the GAN framework. These models are evaluated using other authors’ methods from related work. The evaluation is done using the aforementioned validation set. The ablation study is provided for traditional and Siamese models. At the end of each section, a discussion is presented. In the discussions, comments on achieved results are provided, and open challenges still persist.

#### 3.1.1. Traditional SCNN Classifier Model

As described earlier, a neural network’s first and most common use case is classification. To test the proposed SCNN architecture, an SCNN model, shown in Figure 1, is created. The proposed SCNN model is trained and used to classify chart images in seven chart classes: horizontal bar, vertical bar, line, pie, scatter, horizontal box, and vertical box. The proposed model is trained for 150 epochs using batch size 64 and a learning rate of 0.0005. The parameters are determined empirically during the experiments and considering of our previous work [11,73].

#### 3.1.2. Evaluation

This section evaluates the performance of the proposed SCNN model on the validation dataset. The performance is compared with our previous research [11] and Thiyam et al. [38]. Four evaluation methods are used to conduct comparative experiments including accuracy, precision, recall, and F1 score. Accuracy measures the proportion of correct classifications over all classifications. This is the most common evaluation metric that authors report and should not be mistaken for accuracy calculated using a confusion matrix. Precision measures the percentage of true positives classifications from all classifications that were positive. This research uses a balanced validation dataset, and accuracy and precision are of the same value, although their mathematical equations differ. Recall (sensitivity) measures the percentage of true positive classifications from all classifications that should have been classified as positive. The F1 score is a harmonic mean value of precision and recall. Accuracy, precision, recall, and F1 are formulated as follows:

(1) $$Accuracy=\frac{correct\;classifications}{correct\;classifications+incorrect\;classifications}$$

(2) $$Precision=\frac{true\;positives}{true\;positives+false\;positives}$$

(3) $$Recall=\frac{true\;positives}{true\;positives+false\;negatives}$$

(4) $$F1=2\times \frac{Precision\times Recall}{Precision+Recall}$$

where true positives are positive samples that the model predicted to be positive (e.g., a pie chart is classified as a pie chart), and true negatives where an untrained class is predicted to be negative (e.g., heatmap is not classified into any of trained classes), false positives where a negative sample is predicted to be positive (e.g., a heatmap is classified as a pie chart), and false negatives where a positive sample is predicted as negative by the model (e.g., a pie chart is classified as scatter chart).

**Figure 2:**
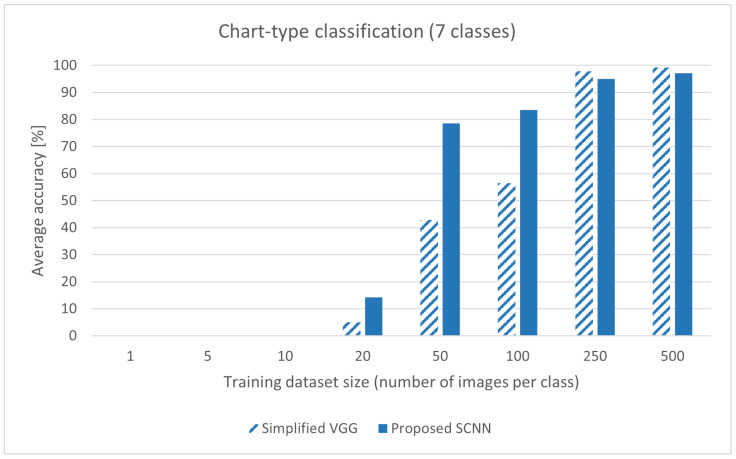
Comparison of average accuracy between a proposed SCNN and a Simplified VGG. The used dataset is ICDAR 2019, with seven chart-type classes. The values are from Table 1.

The detailed comparison results are shown in Table 1. Four evaluation method values are given for 16 different models (eight for proposed SCNN and eight for Simplified VGG). Each model is trained eight times from scratch using the same dataset but with a different number of images. The first column, dataset size per class, refers to the number of images per chart class used for training the neural network (a total of seven chart classes). The table shows that the average accuracy for both architectures is 0% when small datasets are used. Both models cannot learn image features and make correct predictions. By manually inspecting each classified image, it can be seen that the SCNN makes a few correct classifications for a dataset with ten images per class. However, the predicted probability is below 50%, which indicates that the model is unsure in response. When a dataset with 20 images per class was used, the proposed SCNN almost tripled the result of Simplified VGG, but the average accuracy was still low. When datasets with 50 and 100 images per class were used, the proposed SCNN outperforms the Simplified VGG. Using small datasets, the proposed SCNN learns image features fast. However, the rate at which the average accuracy improves between the datasets drops between datasets with 50 and 100 images per class. Increasing the dataset size, the model can learn but at a slower rate compared to a deeper model. The average accuracy from Table 1 is summarized in Figure 2.

To show the architecture complexity of the proposed SCNN, time complexity and space complexity are presented in Table 2. Space complexity is defined by the number of parameters presenting the sum of all weights and biases in the neural network. Max-pooling layer does not contain any number of parameters; instead, it contains hyperparameters. Time complexity includes convolutional layers, max-pooling layers, and fully-connected layers. Convolutional layers occupy 90% of computing time [12]. Multiply-Accumulate operations (MACs) present the time complexity, where one operation includes one multiplication and one addition. Increasing the number of convolution layers, space and time complexity increases significantly. Although different datasets are used in this research and by Thiyam et al. [38], Table 2 compares achieved results in chart classification with different state-of-the-art neural networks. Proposed SCNN achieves competitive results while having the least number of weight layers and time and space complexity. When the proposed SCNN model is compared to Simplified VGG model (both models use the same dataset), the space complexity is reduced by 29 million and time complexity by 0.14 G-operations, negatively impacting the average classification accuracy, causing it to decline by 2.14%.

The statistical comparison of the proposed SCNN model and Simplified VGG model is carried out with McNemar’s test (within-subjects chi-squared test). For calculating a McNemar’s test, data is placed into a 2×2 contingency table where each column contains a number of correct and incorrect predictions. Both models are tested on the same image and the result is noted, e.g., both models predicted correct, one of the models predicted correct, or both models made an incorrect prediction. McNemar’s test calculates whether the Simplified VGG and proposed SCNN agree or disagree in the same way. Table 3 compares all 16 models’ probability values (*p*-values) with a significance level of 0.05. The trained models with datasets of 20, 50, and 100 images per class have a *p*-value less than 0.05, which indicates that null hypothesis H0 can be rejected. The rejected H0 shows that there is a significant difference in the performance of the models. The models make different predictions when introduced to the same image. Although statistical findings do not comment on whether one model is more accurate than the other, they are in correlation with the average accuracy from Figure 2. The proposed SCNN model makes significantly more correct predictions.

#### 3.1.3. Ablation Study

Ablation study is a well-known method used in medicine, where parts of, e.g., the brain are removed to study the effect of further behavior. The same method can be applied to neural networks. In neural networks, an ablation study removes trainable weights and checks the neural network’s performance (classification accuracy) [74]. The goal is to determine which parameters (layers) contribute to the final classification and which can be omitted without decreasing the network’s performance. Performing an ablation study, transparency and interpretability of the network’s behavior are provided. The study also helps to understand how the neural network reaches a decision.

To perform an ablation study, a set of tests for proposed SCNN model are conducted:

- Test-1: disconnected first convolution layer in each convolution block
- Test-2: disconnected second convolution layer in each convolution block
- Test-3: disconnected first convolution block (with max-pooling)
- Test-4: disconnected second convolution block (with max-pooling)
- Test-5: disconnected all max-pooling layers

With the reduction of the number of convolutional layers, the performance of a proposed SCNN model decreases noticeably, Table 4. With the manual inspection of classification results, all tested models find it challenging to classify line and scatter charts. In most test cases, the line and scatter chart are misunderstood or not classified at all. Pie charts achieve the highest classification accuracy. Test 1 and Test 2 provide the same results, as both convolutional layers are built using the same parameters. The only difference is in the second convolution block and second convolution layer, which has spatial padding of 0 px. This test proves that reducing the image by 2 px does not affect final classification accuracy and that the edge of the image does not contain important information. Test 4 achieves the highest classification accuracy of all test cases and indicates that the model performs better when smaller layer channels are used. Disconnecting max-pooling layers (Test 5) significantly increases the time and space complexity of the model, and the model becomes challenging to train (in terms of required time to train and required computational resources).

For the final remarks, it can be concluded that the network’s depth and layer construction play an essential role in learning class-specific features. If the network is too shallow, its learning rate will be noticeably slower, negatively impacting the classification accuracy.

#### 3.1.4. Discussion

Based on the results of the experiments, the proposed SCNN architecture shows both efficiency and accuracy. Compared to other related work, this model gains significantly higher accuracy with much smaller training sets, as shown in Table 2. Also, the computational resources used are significantly lesser. The performed statistical tests confirm that the difference exists between [11] and this work. As expected, the shallow CNN shows somewhat lower performance as the training set size grows compared to a Simplified VGG. However, the vital difference in much higher accuracy and lesser resource consumption with smaller datasets remains. Further reducing the networks’ depth will not yield better performance, as the ablation study showed.

Therefore, it can be concluded that the proposed SCNN architecture is very suitable for use with limited training sets and computational resources.

### 3.2. Siamese SCNN Classifier Model

**Figure 3:**
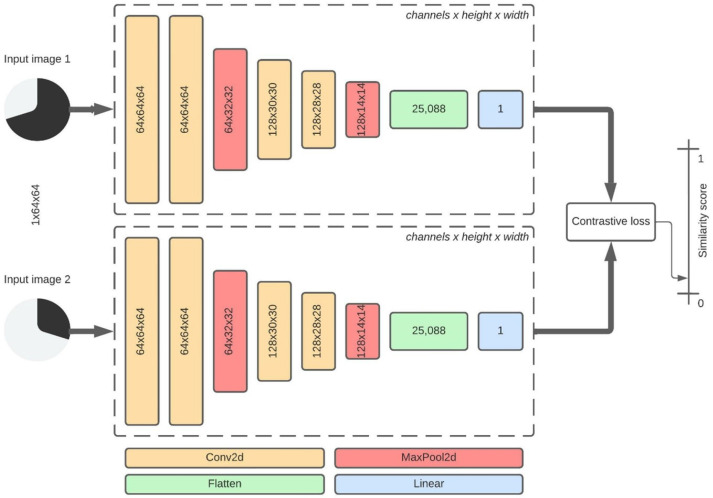
Architecture structure for the proposed Siamese SCNN model. The model consists of two identical proposed SCNN models. The number of weight layers is doubled.

The Siamese SCNN is a subclass of a CNN where neural network architecture is built using two (twin) neural networks. The proposed Siamese SCNN architecture from Figure 3 contains two SCNN models proposed in this research. Both SCNN models are identical, with the same configuration, shared weights, and parameters mirrored and updated in both models. Although the networks use the exact weights, the input image (the input vector) is different, and networks produce different output vectors. One output vector is precomputed and creates a baseline against the other output vector that will be compared. The output vectors will contain the same values if two identical images are introduced to the inputs. These output vectors are used for calculating contrastive loss, a loss function that calculates Euclidean distance between two vectors. Compared to traditional SCNN, which outputs a probability score from 0 to 1, the result of contrastive loss calculation is a similarity score with a value between 0 and 1. For two identical images, the similarity score is 0, and this is the most basic test that verifies the correctness of the model. When two different images, e.g., pie charts, are introduced to the network inputs, the similarity score should be closer to zero, which indicates the same class.

#### 3.2.1. Evaluation

The training parameters of the proposed Siamese SCNN model are the same as for the traditional SCNN model. Still, the evaluation of the Siamese SCNN model differs from the traditional SCNN model since the Siamese SCNN model does not output probability value but instead similarity score. For multi-class classification, a similarity score of the input image needs to be calculated for each of the trained classes. The process for calculating a similarity score for each class is called N-way-K-shot learning, where N is the number of classes and K is the number of samples from each class [75]. In this research, seven-way-one-shot learning is used. One Siamese SCNN input is introduced with a familiar chart image used in the training process. Other Siamese SCNN input is introduced with an image from the validation set. This process creates seven image pairs for one image, e.g., pie & line, line & line, scatter & line, vertical box & line, horizontal box & line, vertical bar & line, horizontal bar & line.

For each image pair, the model calculates a similarity score. If the lowest similarity score in the group is “line & line,” the result is classified as correct (true positive). The process is repeated 20 times; 140 image pairs are tested for one chart class and 980 for all seven classes. With Siamese SCNN models, the dataset’s quality is more important than quantity. When an input image is paired with a random image from each class, a result can be a hit-or-a-miss. If the model chooses a similar image, the similarity score can be close to zero; otherwise, the similarity score will be closer to one. The input image is paired against all trained images to remove an a-hit-or-a-miss effect. The highest similarity images from each class are grouped, and new image pairs for evaluation are created. This method increases the total number of image pairs to 140× number of classes × dataset size. The evaluation results are shown in Table 5 and Table 6, where Table 5 shows an evaluation of input paired with a random image from each class. Table 6 shows the evaluation of input paired with the highest similarity image from each class. Both tables show a comparison with Siamese Simplified VGG model [11].

With small datasets, the network can learn image features. Table 5 shows that the proposed Siamese SCNN model outperforms the Siamese Simplified VGG model. A hit-or-a-miss effect can be seen on a dataset with five images per class for the proposed Siamese SCNN model and on a dataset with 20 images per class for the Siamese Simplified VGG model. In both cases, the accuracy decreases with the increase in training dataset size.

In Table 6, the a-hit-or-a-miss effect is eliminated, and accuracy increases with the dataset size. With the increased dataset size, the network can learn at a slower pace. This phenomenon is also noted when the network is used as a traditional SCNN. On small datasets, the proposed Siamese SCNN model outperforms the Siamese Simplified VGG model as it learns image features fast. Both models achieve identical scores when a dataset with 20 images per class is used. For achieving state-of-the-art accuracy, 100 images per class are needed. The more complex Siamese Simplified VGG model achieves state-of-the-art results with fewer images.

**Figure 4:**
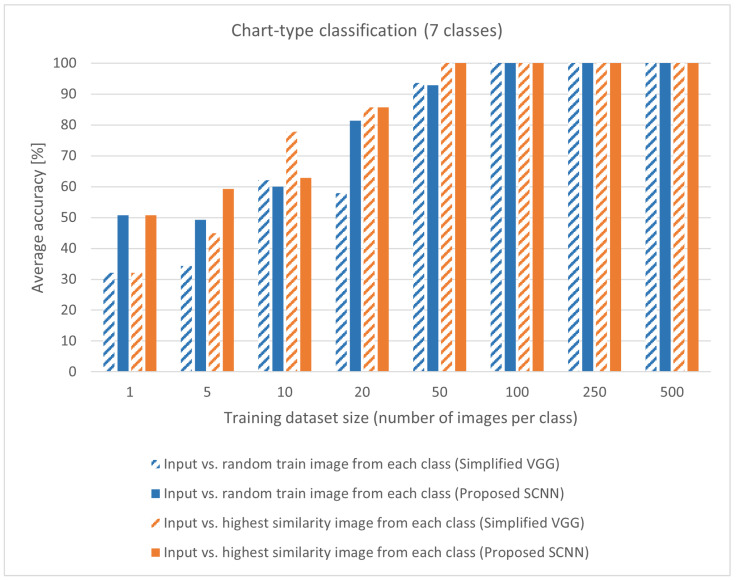
Comparison of average accuracy between a proposed Siamese SCNN model and a Siamese Simplified VGG model. The proposed Siamese SCNN model achieves better classification accuracy than a deeper neural network. The used dataset is ICDAR 2019, with seven chart-type classes. The values are from Table 5 and Table 6.

The accuracy of Table 5 and Table 6 for proposed Siamese SCNN model and Siamese Simplified VGG model is summarized in Figure 4. Both models achieve 100% average accuracy over the same validation set.

The proposed Siamese SCNN model achieves the same accuracy as Siamese Simplified VGG model, using less computational power as shwon in Table 7. When the results of the proposed Siamese SCNN model are compared to Table 2, it is evident that the model outperforms other state-of-the-art neural networks, such as VGG, AlexNet, ResNet, and Inception. To achieve these results, the Siamese network requires ten times fewer images for training. To our knowledge, no other publication classifies chart images using Siamese architecture.

A statistical hypothesis test is conducted using McNemar’s test for statistical comparison of the models. The McNemar’s test is run three times, one time for comparing the results of proposed Siamese SCNN model and two times for comparing the results of proposed Siamese SCNN model with Siamese Simplified VGG model, Table 8. The proposed Siamese SCNN model makes considerably different predictions when small datasets are used and achieves the same performance as larger datasets.The significant difference between the models for the proposed Siamese SCNN model is only where datasets with 5 and 10 images per class are used, and H0 can be rejected. The significant difference for a dataset with one image per class is impossible to achieve since that one image is random and has the highest similarity. When the proposed Siamese SCNN model is compared to the Siamese Simplified VGG model, the H0 can be rejected up to a dataset with 20 images per class in a case when the input image is paired with a random image from each class. The H0 can be rejected up to a dataset with ten images per class for an input image paired with the highest similarity image from each class. This finding correlates with the achieved accuracy in Table 5 and Table 6.

#### 3.2.2. Ablation Study

Complementary to the proposed SCNN model investigation, tests are conducted for the proposed Siamese SCNN model. The results are summarized in Table 9.

Performed tests:

- Test-1: disconnected first convolution layer in each convolution block *
- Test-2: disconnected second convolution layer in each convolution block *
- Test-3: disconnected first convolution block (with max-pooling) *
- Test-4: disconnected second convolution block (with max-pooling) *
- Test-5: disconnected all max-pooling layers *

* (the layers are equally disconnected in both neural networks).

With the reduction of the convolutional layers, the proposed Siamese SCNN model can perform but with average classification accuracy decreased. The removal of convolutional layers reduces the neural network learning rate. Overall, the models with disconnected convolution layers require twice as much dataset size to achieve the same performance as a proposed Siamese SCNN model. Test 1 and Test 2 provide the same results. Between Test 3 and Test 4, the network performs better when smaller layer channels are used. Disconnecting max-pooling layers (Test 5) significantly increases the model’s time and space complexity, contrary to creating a lightweight network. The ablation study results of the proposed Siamese SCNN model are correlated with the ablation study results of a proposed SCNN model. Both models behave equally, which is expected as the proposed Siamese SCNN is built using two proposed SCNN networks.

#### 3.2.3. Discussion

As shown throughout this chapter, the main strength of the Siamese SCNN compared to a traditional SCNN is the ability to perform well with a smaller training set size. Table 5 and Table 6 show that only 100 images per class in the training set are enough for Siamese networks to achieve the maximum accuracy of a 100%. Considering that Siamese SCNN is significantly smaller than the other deep networks, it is much more computationally efficient.

Statistical tests and the ablation study are consistent with the results of the same tests for a classic SCNN. Considering the computational performance and the accuracy gain, it can be concluded that Siamese SCNN brings a further improvement in the field of shallow CNNs.

### 3.3. DCGAN Model

**Figure 5:**
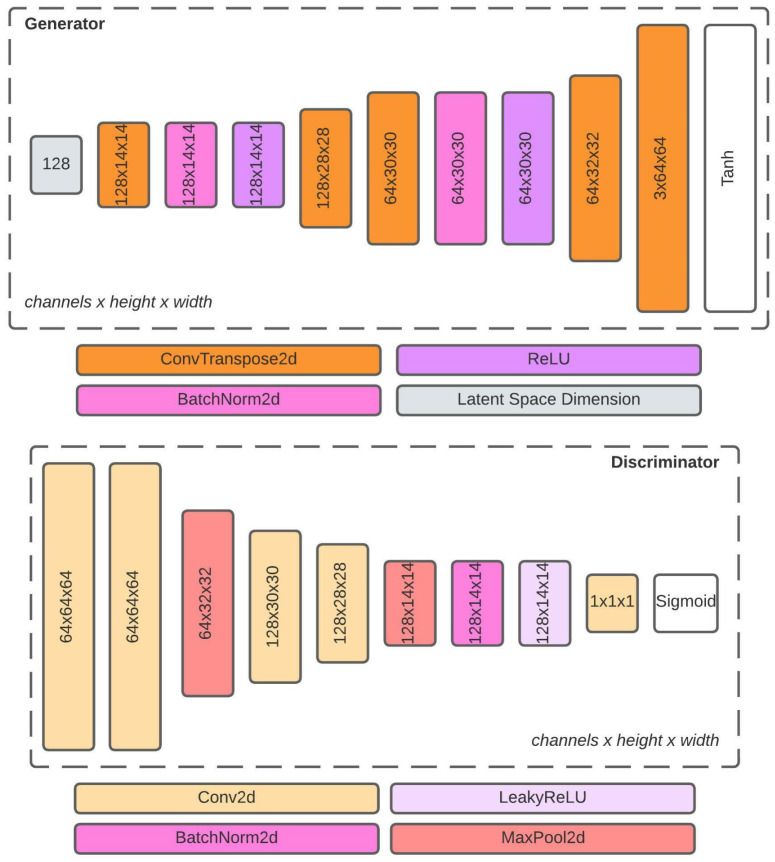
Architecture structure for the proposed DCGAN model. The model uses modified proposed SCNN model for generator and discriminator.

From related work, it can be seen that GANs have successfully generated synthetic images in many different scientific fields. GANs are often challenging to train and stabilize during the training as they are made of fully-connected layers. To tackle the challenge, DCGANs are the most widely used. Encouraged by the DCGAN’s success and ability to create quality images, a DCGAN architecture is proposed in Figure 5. The architecture consists of two distinct models, a generator and a discriminator. Both models are trained simultaneously using an adversarial process. A discriminator is seen as a traditional CNN, a predictive model that outputs probability and classifies input images into two categories. A primary task of the discriminator model is to notice whether the input image belongs to the group of natural images from the real dataset, or the input image is fake and generated by the generator model.

On the other hand, the generator’s main task is to create images that look like they are from the training set. During the model training process, the generator improves at creating fake images, while the discriminator improves at classifying natural and fake images. The two models compete to get a better result. The perfect result of competition is when the generator creates images that look like they are from the training set and when the discriminator’s probability outcome is at 50%, meaning that the discriminator is unsure whether the image is natural or fake.

The generator model from Figure 5 receives an input, a random noise, or a 128-dimensional latent space vector. The choice of a 128-dimensional latent space vector is inspired by Marin et al. [76]. The author explored different latent space dimensions, such as 4, 16, 100, 128, and 512, on a large-scale dataset of celebrity faces, and achieved the best qualitative and quantitative evaluation results using a 128-dimensional latent space vector. The random noise is inputted to the convolutional transpose layer. The convolutional transpose layer performs inverse convolution operation. It upsamples a small input image into a larger output image. In the first convolutional transpose layer a 14×14 receptive field filter is used with a convolutional stride fixed to 1 px, and spatial padding of 0 px. The sizeable receptive field filter reduces the depth of the network and the number of details that will be upsampled. When using small size receptive field filters, salt, and papper noise occurred on final images. The convolutional transpose layer is followed by batch normalization and a Rectified Linear Unit (ReLU) activation function. Batch normalization is used to accelerate the convergence speed of the model and stabilize the learning process. The ReLU activation function allows the model to learn more quickly and to cover the color space. Batch normalization and a ReLU activation function are used after each convolutional block, except the last, where the hyperbolic tangent (Tanh) activation function is used. The first convolutional block consists of two convolutional transpose layers with different parameters. The first convolutional transpose layer uses a 4×4 receptive field filter size with a convolutional stride fixed to 2 px and spatial padding set to 1 px. The result is an image with dimensions doubled. The second convolutional transpose layer uses a 3×3 receptive field filter size with a convolutional stride fixed to 2 px and spatial padding set to 1 px. The second convolutional block uses the same combination of receptive field filters, convolutional strides, and spatial paddings as the first convolutional block. The result is an image with 64×64 px dimensions and red-green-blue color space.

The discriminator model uses the same combination of convolutional blocks as a proposed SCNN architecture. The difference is in the last part of the architecture, where a fully-connected layer is replaced with batch normalization and a Leaky ReLU. The batch’s normalization main task is the same as in the generator model. Leaky ReLU replaces the ReLU activation function of the generator model. The Leaky ReLU activation function prevents a dying state phenomenon; that is, a ReLU activation function outputs zero values instead the maximum values between an input value and zero. The Leaky ReLU activation function allows the passing of a slight gradient of negative values. These gradients are passed into a generator model, which enables a better learning process and higher quality output images. At the end of a model, instead of softmax operation, which is used for multi-class classification, a sigmoid operation is used for binary classification.

#### 3.3.1. Evaluation

The training of GANs is challenging as two models compete to achieve better performance. The performance improvement of one model often results in a decrease in the performance of the other model. The training of a model can also result in failures that can be classified as:

- Mode Collapse (MC), a model that cannot produce a variety of input data. The model usually outputs a single image or a similar set of a few images. MC is visually hard to notice when there is a larger size training dataset, but with smaller dataset sizes is easier noticeable. The generated images can be high quality, and manual inspection is required. During the training process, MC can be noticed when generator loss takes on a high value, and a discriminator loss stays around zero.
- Convergence Failure (CF), a model that produces poor-quality images. The output images lack color or are filled with salt and papper noise. During the training process, CF can be noticed when generator loss and discriminator loss do not move from zero, or when generator loss or discriminator loss takes on an extremely high value and the other does not move from zero.

To quantitatively evaluate a proposed DCGAN model, FID [77] and IS [78] scores are used. Both metrics use a pre-trained Inception model to measure a feature distance between images. The IS score correlates with human judgment. Performance measuring uses the generated images’ quality and diversity. The IS does not capture how generated images compare to the natural images. The higher the value of IS score the images are of better quality.

On the other hand, FID calculates Gaussian distribution with mean and covariance between natural and generated images, also known as Wasserstein-2 distance. The score captures the similarity between natural and generated images. Contrary to IS, lower values of FID indicate images of better quality and diversity.

**Figure 6:**
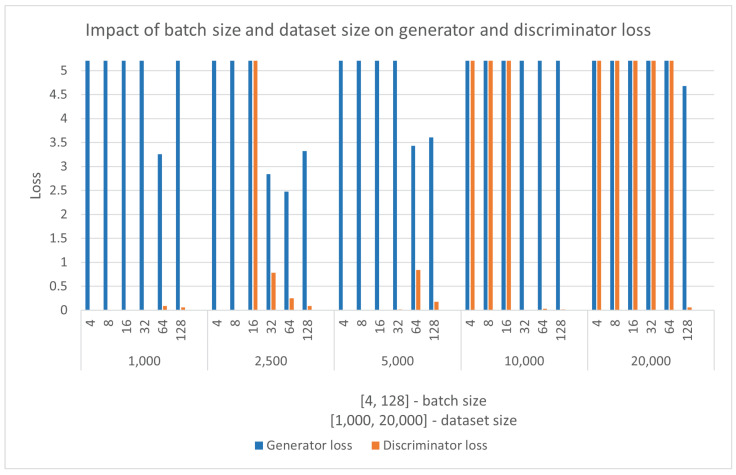
Impact of batch size and dataset size on generator and discriminator. A stable DCGAN model has a discriminator loss of around 0.5 and a generator loss of up to 4. All models are trained for 150 epochs.

The GANs are sensitive to hyperparameters, and finding a combination that will not result in MC or CF is challenging. In Figure 6, dataset size and batch size are compared to generator and discriminator loss during the training process. The proposed DCGAN model is trained from scratch six times for each dataset size, resulting in 30 different models. It is essential to note that the refereed dataset size contains images of a single chart class. The process is repeated for multiple chart classes, and the results do not significantly change. To better understand Figure 6, Table 10 is provided with summarized results.

**Figure 7:**
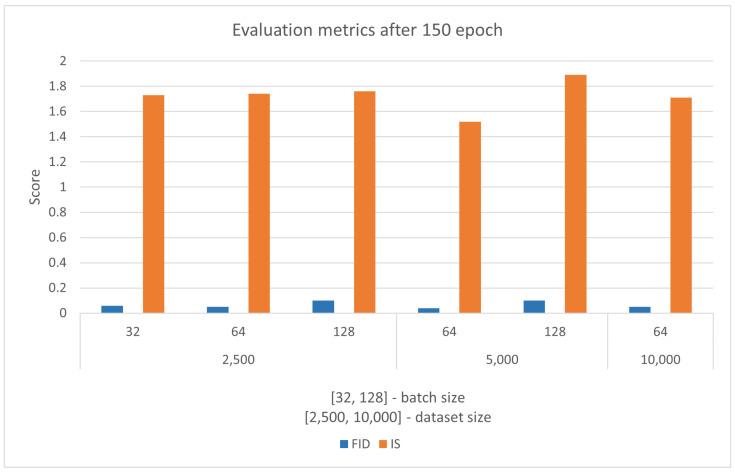
FID and IS scores for six stable DCGAN models from Figure 6. The performance of all six models is similar, but with manual inspection, the best performing model is trained with 2500 images and batch size 32. The worst performing model is trained with 5000 images and 128 batch size.

Figure 6 and Table 10 show that only six models do not result in a training failure. All training of the proposed model is done over 150 epochs. The results show that the proposed DCGAN model does not produce stable output when small and large dataset sizes are used with any batch size. A stable DCGAN model has a discriminator loss of around 0.5 and a generator loss of up to 4. All model outputs are manually inspected. It was established that although a model that uses a 1000 image dataset and 64 batch size meet the criteria of a stable DCGAN, the output results with MC. With manual inspection, a model that uses a 10,000 image dataset and 64 batch size, by the discriminator and generator score, looks like an MC, but instead, it produces a stable output. The six successful models are evaluated using FID and IS scores, and the results are presented in Figure 7.

**Figure 8:**
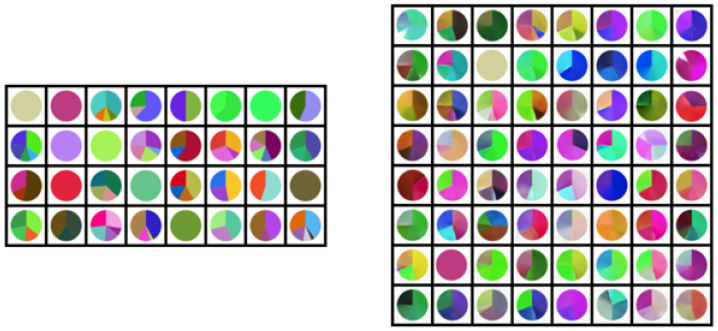
The comparison of natural images and generated images. On the left is one batch with 32 natural images used in the training process. On the right side are 64 images generated by the proposed DCGAN. The used DCGAN model is trained with 2500 images and 32 batch size.

The results of all six models are very similar, although different datasets and batch sizes are used. Judging by Figure 7, it is hard to notice which model performs the best and generates the images of the highest quality. With the manual inspection of generated mages, the difference in quality is noticeable. With objective judgment, the model trained with 2500 images and batch size 32 has the highest quality and diversity of generated images. The model trained with 5000 images and batch size 64 has similar image quality. The worst performing model is trained with 5000 images and 128 batch size. The results of the best-performing model are shown in Figure 8.

On the left is one batch with 32 natural images used in the training process. On the right side are 64 images generated by the proposed DCGAN. On the generated images, a few things should be noticed:

Positive:

- The background is always white and does not contain salt and papper noise
- The circular shape is always present
- The chart images contain single and multi-color
- The diversity of used colors is present
- Different proportions of different and same colors are present

Negative:

- The boundary where colors meet is unclear and dimly
- When white color is used, the edges are hard to notice

**Figure 9:**
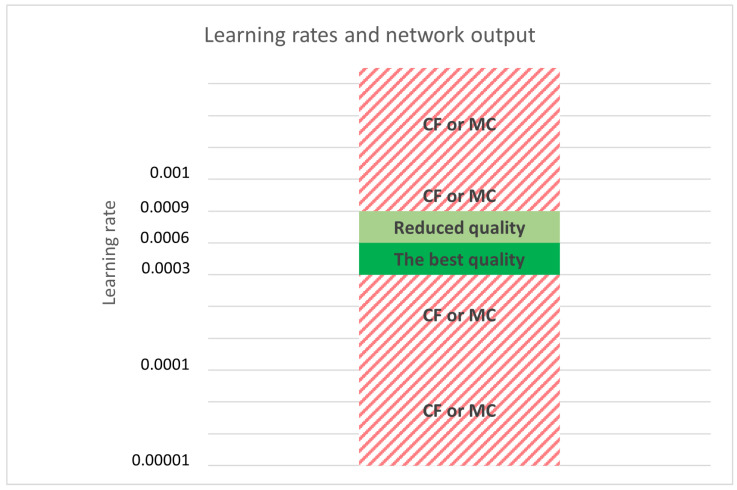
Impact of learning rate on DCGAN output. Multiple learning rates are tested. The proposed model is functional with any learning rate between 0.0003 and 0.0009. The best output quality is achieved with a learning rate between 0.0003 and 0.0006.

DCGANs are also sensitive to learning rate hyperparameters. Changing a learning rate without changing the generator’s or discriminator’s architecture significantly impacts the output of the DCGAN model. Figure 9 shows all tested learning rate values used with all models mentioned above. The results show that the highest image quality and diversity are achieved when the learning rate is between 0.0003 and 0.0006. The proposed model is functional with any learning rate between 0.0003 and 0.0009. Any higher or lower value results with MC or CF. The generator and discriminator do not have to have the same learning rate, and the model that generated the images in Figure 8 uses a learning rate of 0.0003 for the generator and a learning rate of 0.0004 for the discriminator; in other words, the generator learns slower with attention to detail.

#### 3.3.2. Discussion

In this research, a proposed SCNN architecture is modified and used as a generator and discriminator in the DCGAN model. An input of a generator is a 128-dimensional latent space vector which transforms to an image with 64×64 px dimensions and red-green-blue color space. Since DCGANs are challenging to train, multiple-size datasets and batch sizes are tested according to their instability. The dataset sizes range from 1000 to 20,000 images per chart class, and batch sizes range from 4 to 128. The impact on learning rate is also inspected, and multiple values are tested.

The proposed DCGAN model has been trained from scratch more than 100 times, and only six models are found to be stable. These six models are evaluated using FID and IS scores. To give a conclusion based on FID and IS scores is challenging, as all six models achieve similar values. Using manual inspection of generated images, it is decided that the best performing model, in terms of image quality and diversity, is a model trained with 2500 images, using a batch size of 32 and a learning rate for generator of 0.0003 and discriminator of 0.0004. The evaluation of the model using FID achieved a score of 0.06 (the lower, the better) and using IS score of 1.73 (the higher, the better). The presented scores indicate that the generated images are close to the original, but improvements can be made. A better balance between a generator and a discriminator should be found in future research. A generator must be strengthened by adding convolutional transpose layers at the network’s input. With added new layers, the receptive field filter will be reduced, and the model should be able to learn better the boundary between colors. In previous sections, an ablation study is done for each neural network model. Excluding layers from DCGAN results in high instability of the model, and all hyperparameters need to be changed accordingly. The number of potential models for testing grows exponentially, and with the limited computational resources, this becomes out of the scope of the presented research. To our knowledge, no other research uses the DCGAN model to generate chart images, and the results present a good starting point for future research.

## 4. Conclusions

This paper presents a new multi-purpose SCNN architecture for chart images. The primary network model consists of four convolutional layers, two max-pooling layers, and one fully-connected layer. To present the abilities of this architecture, three use cases related to chart images are explored: traditional CNN, Siamese CNN, and a GAN (DCGAN) for the generation of chart images.

When used as a traditional CNN, SCNN was shown to be extremely useful. It achieved a very high average accuracy (above 97%) with only a fraction of the complexity of competitors (Table 2). Also, it is shown that the proposed SCNN learns much faster than the nearest competitor, Simplified VGG. The correctness of this model was confirmed with McNemar’s test and the ablation study.

The performance of the proposed SCNN was further improved when it was used as a Siamese CNN. Namely, the main strength of the Siamese SCNN compared to a plain SCNN is the ability to perform well with a smaller training set size. In the experiments, only 100 images per class in the training set were enough for Siamese networks to achieve the maximum average accuracy of 100%. The correctness of this model was also confirmed with tests. Given the computational performance and the accuracy, it is clear that the Siamese SCNN model is a further improvement of SCNNs.

The proposed SCNN architecture was also modified and used as a generator and discriminator in the DCGAN model. The biggest challenge was to train a DCGAN. Therefore multiple-size datasets and batch sizes were tested. The proposed DCGAN model was trained from scratch more than 100 times, and only six models were found to be stable. Out of those six, the best-performing model, in terms of image quality and diversity, was chosen, trained, and evaluated. The presented results show that the generated images are close to the original, yet some improvements could be made. To our knowledge, this is the first application of the DCGAN model in chart image generation.

In conclusion, this paper presented a novel SCNN architecture and showed its value in three different use cases. The results confirm that SCNNs should be considered a viable solution to many CNN applications, not only for chart image classification. SCNNs prove to be a powerful tool for chart image classification and generation, comparable in results with DCNNs, but much more efficient.

Future research would focus on improving DCGAN as a chart image generator. This includes better balancing between a generator and a discriminator and strengthening the generator by adding convolutional transpose layers at the network’s input. For Siamese SCNN, other loss functions should be tested (triplet loss, magnet loss, center loss). The plan is also to increase the number of supported chart-type classes. Since the research is based on synthetic chart images, the performance should be tested on real-world chart images.

## Figures and Tables

**Table 1:** Performance comparison of a proposed SCNN and a Simplified VGG over eight different datasets. The proposed SCNN achieves better performance on smaller size datasets. Increasing the dataset size, the proposed SCNN is able to learn but at a slower rate compared to a deeper neural network.

Dataset Size per Class	Proposed SCNN				Simplified VGG [11]			
	Accuracy	Precision	Recall	F1	Accuracy	Precision	Recall	F1
1	0	0	0	0	0	0	0	0
5	0	0	0	0	0	0	0	0
10	0	0	0	0	0	0	0	0
20	14.28	14.28	19.64	16.18	5	5	15.99	5.85
50	78.57	78.57	81	78.38	42.85	42.85	48.22	44.87
100	83.57	83.57	82.84	83.03	56.42	56.42	57.7	56.63
250	95	95	95.13	94.98	97.85	97.85	98.7	98.56
500	97.14	97.14	97.4	97.13	99.28	99.28	99.32	99.28

**Table 2:** Different neural networks are used for chart-type classification. The neural networks are built using weight layers and described with time and space complexity. The proposed SCNN model has the least number of weight layers and the lowest time and space complexity. The achieved results are competitive with complex neural networks.

Architecture	Parameters (M)	MACs (G)	Weight Layers ↓	Training Dataset Size	Accuracy (%)
Proposed SCNN	0.5	0.33	5	3500	97.14
Simplified VGG [11]	29.78	0.47	7	3500	99.28
AlexNet [33]	61.1	0.77	8	45,617	95.17
VGG16 [33]	138.36	15.61	16	45,617	97.44
VGG19 [33]	143.67	19.77	19	45,617	95.78
Inception v3 [33]	27.16	5.75	48	45,617	83.32
ResNet50 [33]	25.56	4.14	50	45,617	85.71
ResNet101 [33]	44.55	7.87	101	45,617	87.59
ResNet152 [33]	60.19	11.61	152	45,617	87.47

**Table 3:** The McNemar’s test with significance level of 0.05. The results are in accordance with the classification results from Table 1 and Figure 2.

Dataset Size per Class	Proposed SCNN	
	*p* < 0.05	H0
1	false	failed
5	false	failed
10	false	failed
20	true	reject
50	true	reject
100	true	reject
250	false	failed
500	false	failed

**Table 4:** Results of ablation study for proposed SCNN model. The reduction of weight layers decreases the performance noticeably.

Dataset Size per Class	Proposed Siamese SCNN	Test-1, Test-2	Test-3	Test-4
1	0	0	0	0
5	0	0	0	0
10	0	0	0	0
20	14.28	12.85	8.57	11.42
50	78.57	42.14	34.28	40
100	83.57	62.85	74.28	77.85
250	95	73.57	83.57	86.42
500	97.14	87.14	85	90

**Table 5:** Performance comparison of a proposed Siamese SCNN model and a Siamese Simplified VGG model over eight different datasets using random image from each class. The proposed Siamese SCNN model achieves the same classification accuracy as a deeper model. A performance decrease when dataset size is increasing is called a hit-or-a-miss effect.

Input vs. Random Image from Each Class								
**Dataset** **Size per Class**	**Proposed Siamese SCNN**				**Siamese Simplified VGG [11]**			
	**Accuracy**	**Precision**	**Recall**	**F1**	**Accuracy**	**Precision**	**Recall**	**F1**
1	50.71	50.71	53.38	51.28	32.14	32.14	40.46	32.45
5	49.28	49.28	49.85	49.24	34.28	34.28	41.47	35.36
10	60	60	63.32	59.79	62.14	62.14	64.39	62.53
20	81.42	81.42	81.13	81.24	57.85	57.85	60.65	57.45
50	92.85	92.85	93.05	92.87	93.57	93.57	94.28	94.28
100	100	100	100	100	100	100	100	100
250	100	100	100	100	100	100	100	100
500	100	100	100	100	100	100	100	100

**Table 6:** Performance comparison of a proposed Siamese SCNN model and a Siamese Simplified VGG model over eight different datasets using the highest similarity image from each class. The proposed Siamese SCNN model learns more image features with smaller datasets, but with the increased dataset size, the learning continues at a slower pace. Both networks achieve state-of-the-art results in chart-type classification.

Input vs. Highest Similarity Image from Each Class								
**Dataset** **Size per Class**	**Proposed Siamese SCNN**				**Siamese Simplified VGG [11]**			
	**Accuracy**	**Precision**	**Recall**	**F1**	**Accuracy**	**Precision**	**Recall**	**F1**
1	50.71	50.71	53.38	51.28	32.14	32.14	40.46	32.45
5	59.28	59.28	59.79	59.24	45	45	49.3	46.1
10	62.85	62.85	65.78	62.38	77.85	77.85	78.91	78.66
20	85.71	85.71	86.08	85.67	85.71	85.71	86.6	85.62
50	92.85	92.85	93.03	92.81	100	100	100	100
100	100	100	100	100	100	100	100	100
250	100	100	100	100	100	100	100	100
500	100	100	100	100	100	100	100	100

**Table 7:** Only one previous work focuses on chart-type classification using a Siamese neural network. The proposed Siamese SCNN model achieves state-of-the-art results on the same dataset using fewer computational resources.

Architecture	Parameters (M)	MACs (G)	Weight Layers ↓	Training Dataset Size	Accuracy (%)
Proposed Siamese SCNN	1.01	0.67	10	3500	100
Siamese Simplified VGG [11]	59.56	0.94	14	3500	100

**Table 8:** The McNemar’s test with a significance level of 0.05. The table shows three tests for the proposed Siamese SCNN model. The H0 can be rejected for smaller-size datasets. The results are in accordance with the classification results from Table 5 and Table 6.

Dataset Size per Class	Proposed Siamese SCNN		Proposed Siamese SCNN—Siamese Simplified VGG [11]			
	Input vs. Random Image from Each Class— Input vs. Highest Similarity Image from Each Class		Input vs. Random Image from Each Class		Input vs. Highest Similarity Image from Each Class	
	*p* < 0.05	H0	*p* < 0.05	H0	*p* < 0.05	H0
1	false	failed	true	reject	true	reject
5	true	reject	true	reject	true	reject
10	true	reject	true	reject	true	reject
20	false	failed	true	reject	false	failed
50	false	failed	false	failed	false	failed
100	false	failed	false	failed	false	failed
250	false	failed	false	failed	false	failed
500	false	failed	false	failed	false	failed

**Table 9:** Results of ablation study for proposed Siamese SCNN model. The reduction of weight layers decreases the performance noticeably.

Dataset Size per Class	Average Accuracy (%)							
	Input vs. Random Image from Each Class				Input vs. Highest Similarity Image from Each Class			
	Proposed Siamese SCNN	Test-1, Test-2	Test-3	Test-4	Proposed Siamese SCNN	Test-1, Test-2	Test-3	Test-4
1	50.71	50	45.71	42.85	50.71	50	45.71	42.85
5	49.28	53.57	28.57	55	59.28	54.28	51.42	63.57
10	60	52.14	57.14	56.42	62.85	60	65.71	71.42
20	81.42	85	75	79.28	85.71	85	80.71	83.57
50	92.85	81.42	86.42	93.57	92.85	91.42	86.42	95
100	100	92.85	93.57	97.14	100	93.57	94.28	97.85
250	100	95	96.42	100	100	100	98.57	100
500	100	100	100	100	100	100	100	100

**Table 10:** Summary of Figure 6 with a type of mode failure; Mode Collapse (MC), Convergence Failure (CF).

Batch Size	Dataset Size				
	1000	2500	5000	10,000	20,000
4	CF	CF	CF	CF	CF
8	CF	CF	CF	CF	CF
16	CF	CF	CF	CF	CF
32	CF		MC	CF	CF
64	MC				CF
128	MC			MC	MC

## Data Availability

Not applicable.

## Abbreviations

The following abbreviations are used in this manuscript:

ANNs	Artificial Neural Networks
CNNs	Convolutional Neural Networks
DCNNs	Deep Convolutional Neural Networks
SCNN	Shallow Convolutional Neural Networks
GAN	Generative Adversarial Networks
SVMs	Support Vector Machines
WGAN	Wasserstein Generative Adversarial Network
LSGAN	Least Squares Generative Adversarial Network
DiscoGan	Disco Generative Adversarial Network
CycleGAN	Cycle-Consistent Generative Adversarial Network
FID	Frechet Inception Distance
IS	Inception Score

## References

[1] Friendly M., Chen C.H., Härdle W., Unwin A. (2008). A Brief History of Data Visualization. Handbook of Data Visualization.

[2] Jensen C., Anderson L. (1992). Harvard Graphics: The Complete Reference.

[3] Davila K., Kota B.U., Setlur S., Govindaraju V., Tensmeyer C., Shekhar S., Chaudhry R. ICDAR 2019 Competition on Harvesting Raw Tables from Infographics (CHART-Infographics). Proceedings of the 2019 International Conference on Document Analysis and Recognition (ICDAR).

[4] Poco J., Heer J. (2017). Reverse-Engineering Visualizations: Recovering Visual Encodings from Chart Images. Comput. Graph. Forum.

[5] Wang J., Luo C., Huang H., Zhao H., Wang S. (2017). Transferring Pre-Trained Deep CNNs for Remote Scene Classification with General Features Learned from Linear PCA Network. Remote Sens..

[6] Lecun Y., Bottou L., Bengio Y., Haffner P. (1998). Gradient-based learning applied to document recognition. Proc. IEEE.

[7] Krizhevsky A., Sutskever I., Hinton G.E. (2017). ImageNet classification with deep convolutional neural networks. Commun. ACM.

[8] Simonyan K., Zisserman A. (2014). Very Deep Convolutional Networks for Large-Scale Image Recognition. arXiv.

[9] Szegedy C., Liu W., Jia Y., Sermanet P., Reed S., Anguelov D., Erhan D., Vanhoucke V., Rabinovich A. Going deeper with convolutions. Proceedings of the 2015 IEEE Conference on Computer Vision and Pattern Recognition (CVPR).

[10] He K., Zhang X., Ren S., Sun J. Deep Residual Learning for Image Recognition. Proceedings of the 2016 IEEE Conference on Computer Vision and Pattern Recognition (CVPR).

[11] Bajić F., Job J. (2021). Chart Classification Using Siamese CNN. J. Imaging.

[12] Lei F., Liu X., Dai Q., Ling B.W.K. (2020). Shallow convolutional neural network for image classification. SN Appl. Sci..

[13] Zhou Y.P., Tan C.L. Hough technique for bar charts detection and recognition in document images. Proceedings of the 2000 International Conference on Image Processing (Cat. No.00CH37101).

[14] Zhou Y.P., Tan C.L., Anderson M., Cheng P., Haarslev V. (2000). Bar Charts Recognition Using Hough Based Syntactic Segmentation. Theory and Application of Diagrams.

[15] Redeke I. Image & Graphic Reader. Proceedings of the 2001 International Conference on Image Processing (Cat. No.01CH37205).

[16] Gao J., Zhou Y., Barner K.E. View: Visual Information Extraction Widget for improving chart images accessibility. Proceedings of the 2012 19th IEEE International Conference on Image Processing.

[17] Beagle: Automated Extraction and Interpretation of Visualizations from the Web. Proceedings of the 2018 CHI Conference on Human Factors in Computing Systems.

[18] Mishra P., Kumar S., Chaube M.K. (2021). ChartFuse: A novel fusion method for chart classification using heterogeneous microstructures. Multimed. Tools Appl..

[19] Mishchenko A., Vassilieva N. (2011). Model-Based Recognition and Extraction of Information from Chart Images. J. Multim. Process. Technol..

[20] Mishchenko A., Vassilieva N., Bebis G., Boyle R., Parvin B., Koracin D., Wang S., Kyungnam K., Benes B., Moreland K., Borst C., DiVerdi S. (2011). Model-Based Chart Image Classification. Advances in Visual Computing.

[21] ReVision: Automated Classification, Analysis and Redesign of Chart Images. Proceedings of the 24th Annual ACM Symposium on User Interface Software and Technology.

[22] Nair R.R., Sankaran N., Nwogu I., Govindaraju V. Automated analysis of line plots in documents. Proceedings of the 2015 13th International Conference on Document Analysis and Recognition (ICDAR).

[23] Shi Y., Wei Y., Wu T., Liu Q. Statistical graph classification in intelligent mathematics problem solving system for high school student. Proceedings of the 2017 12th International Conference on Computer Science and Education (ICCSE).

[24] Lin A.Y., Ford J., Adar E., Hecht B. VizByWiki: Mining Data Visualizations from the Web to Enrich News Articles. Proceedings of the 2018 World Wide Web Conference, International World Wide Web Conferences Steering Committee, WWW’18.

[25] Choi J., Jung S., Park D.G., Choo J., Elmqvist N. (2019). Visualizing for the Non-Visual: Enabling the Visually Impaired to Use Visualization. Comput. Graph. Forum.

[26] Jobin K.V., Mondal A., Jawahar C.V. DocFigure: A Dataset for Scientific Document Figure Classification. Proceedings of the 2019 International Conference on Document Analysis and Recognition Workshops (ICDARW).

[27] Kaur P., Kiesel D. Combining Image and Caption Analysis for Classifying Charts in Biodiversity Texts. Proceedings of the 15th International Joint Conference on Computer Vision, Imaging and Computer Graphics Theory and Applications—IVAPP.

[28] Chagas P., Akiyama R., Meiguins A., Santos C., Saraiva F., Meiguins B., Morais J. Evaluation of Convolutional Neural Network Architectures for Chart Image Classification. Proceedings of the 2018 International Joint Conference on Neural Networks (IJCNN).

[29] Kosemen C., Birant D. (2020). Multi-label classification of line chart images using convolutional neural networks. SN Appl. Sci..

[30] Ishihara T., Morita K., Shirai N.C., Wakabayashi T., Ohyama W., Palaiahnakote S., Sanniti di Baja G., Wang L., Yan W.Q. (2020). Chart-Type Classification Using Convolutional Neural Network for Scholarly Figures. Pattern Recognition.

[31] Dadhich K., Daggubati S., Sreevalsan-Nair J. (2022). BarChartAnalyzer: Data Extraction and Summarization of Bar Charts from Images. SN Comput. Sci..

[32] Deng J., Dong W., Socher R., Li L.J., Li K., Fei-Fei L. ImageNet: A large-scale hierarchical image database. Proceedings of the 2009 IEEE Conference on Computer Vision and Pattern Recognition.

[33] Siegel N., Horvitz Z., Levin R., Divvala S., Farhadi A., Leibe B., Matas J., Sebe N., Welling M. (2016). FigureSeer: Parsing Result-Figures in Research Papers. Computer Vision—ECCV 2016.

[34] Balaji A., Ramanathan T., Sonathi V. (2018). Chart-Text: A Fully Automated Chart Image Descriptor. arXiv.

[35] Huang S., Greven S., Wang W. (2020). An Image Classification Tool of Wikimedia Commons.

[36] Araujo T., Chagas P., Alves J., Santos C., Sousa Santos B., Serique Meiguins B. (2020). A Real-World Approach on the Problem of Chart Recognition Using Classification, Detection and Perspective Correction. Sensors.

[37] Dai W., Wang M., Niu Z., Zhang J. (2018). Chart decoder: Generating textual and numeric information from chart images automatically. J. Vis. Lang. Comput..

[38] Thiyam J., Singh S.R., Bora P.K. Challenges in chart image classification: A comparative study of different deep learning methods. Proceedings of the 21st ACM Symposium on Document Engineering, DocEng’21.

[39] Davila K., Tensmeyer C., Shekhar C., Singh H., Setlur S., Govindaraju V. (2021). Competition on Harvesting Raw Tables from Infographics. Pattern Recognition. ICPR International Workshops and Challenges. Lecture Notes in Computer Science.

[40] Li J., Xie S., Chen Z., Liu H., Kang J., Fan Z., Li W. (2020). A Shallow Convolutional Neural Network for Apple Classification. IEEE Access.

[41] Xiao H., Rasul K., Vollgraf R. (2017). Fashion-MNIST: A Novel Image Dataset for Benchmarking Machine Learning Algorithms. arXiv.

[42] Gorokhovatskyi O., Peredrii O. Shallow Convolutional Neural Networks for Pattern Recognition Problems. Proceedings of the 2018 IEEE Second International Conference on Data Stream Mining & Processing (DSMP).

[43] Wang L., Chen A., Zhang Y., Wang X., Zhang Y., Shen Q., Xue Y. (2020). AK-DL: A Shallow Neural Network Model for Diagnosing Actinic Keratosis with Better Performance than Deep Neural Networks. Diagnostics.

[44] Reasat T., Shahnaz C. Detection of inferior myocardial infarction using shallow convolutional neural networks. Proceedings of the 2017 IEEE Region 10 Humanitarian Technology Conference (R10-HTC).

[45] Singh S.P., Wang L., Gupta S., Gulyás B., Padmanabhan P. (2021). Shallow 3D CNN for Detecting Acute Brain Hemorrhage From Medical Imaging Sensors. IEEE Sensors J..

[46] Chen W., Yang B., Li J., Wang J. (2020). An Approach to Detecting Diabetic Retinopathy Based on Integrated Shallow Convolutional Neural Networks. IEEE Access.

[47] Jain A., Singh S.K., Singh K.P. (2020). Handwritten signature verification using shallow convolutional neural network. Multimed. Tools Appl..

[48] Golovko V.A., Egor M., Brich A., Sachenko A. A Shallow Convolutional Neural Network for Accurate Handwritten Digits Classification. Proceedings of the ICPR 2016.

[49] Mukherjee H., Ghosh S., Dhar A., Obaidullah S.M., Santosh K.C., Roy K. (2021). Shallow Convolutional Neural Network for COVID-19 Outbreak Screening Using Chest X-rays. Cogn. Comput..

[50] Huang W., Zhang L., Gao W., Min F., He J. (2021). Shallow Convolutional Neural Networks for Human Activity Recognition Using Wearable Sensors. IEEE Trans. Instrum. Meas..

[51] Sharma A.K., Kang B., Kim K.K. LightNet: A Lightweight Neural Network for Image Classification. Proceedings of the 2021 18th International SoC Design Conference (ISOCC).

[52] Qin N., Liu L., Huang D., Wu B., Zhang Z. (2021). LeanNet: An Efficient Convolutional Neural Network for Digital Number Recognition in Industrial Products. Sensors.

[53] Akiyama R., Araújo T.D., Chagas P., Miranda B., Santos C., Morais J., Meiguins B. Synthetic Chart Image Generator: An Application for Generating Chart Image Datasets. Proceedings of the 2018 22nd International Conference Information Visualisation (IV).

[54] Goodfellow I.J., Pouget-Abadie J., Mirza M., Xu B., Warde-Farley D., Ozair S., Courville A., Bengio Y. (2014). Generative Adversarial Networks. arXiv.

[55] Radford A., Metz L., Chintala S. (2016). Unsupervised Representation Learning with Deep Convolutional Generative Adversarial Networks. arXiv.

[56] Arjovsky M., Chintala S., Bottou L. Wasserstein Generative Adversarial Networks. Proceedings of the 34th International Conference on Machine Learning.

[57] Mao X., Li Q., Xie H., Lau R.Y., Wang Z., Smolley S.P. Least Squares Generative Adversarial Networks. Proceedings of the 2017 IEEE International Conference on Computer Vision (ICCV).

[58] Kim T., Cha M., Kim H., Lee J.K., Kim J. (2017). Learning to Discover Cross-Domain Relations with Generative Adversarial Networks. arXiv.

[59] Zhu J.Y., Park T., Isola P., Efros A.A. (2020). Unpaired Image-to-Image Translation using Cycle-Consistent Adversarial Networks. arXiv.

[60] Venu S.K. (2021). Evaluation of Deep Convolutional Generative Adversarial Networks for data augmentation of chest X-ray images. Future Internet.

[61] Puttagunta M.K., Subban R., C N.K.B. (2022). A Novel COVID-19 Detection Model Based on DCGAN and Deep Transfer Learning. Procedia Comput. Sci..

[62] Xu Z., Wang R.F., Wang J., Yu D.H. (2020). Parkinson’s Disease Detection Based on Spectrogram-Deep Convolutional Generative Adversarial Network Sample Augmentation. IEEE Access.

[63] Alrashedy H.H.N., Almansour A.F., Ibrahim D.M., Hammoudeh M.A.A. (2022). BrainGAN: Brain MRI Image Generation and Classification Framework Using GAN Architectures and CNN Models. Sensors.

[64] Gao H., Zhang Y., Lv W., Yin J., Qasim T., Wang D. (2022). A Deep Convolutional Generative Adversarial Networks-Based Method for Defect Detection in Small Sample Industrial Parts Images. Appl. Sci..

[65] Zhang Y., Wa S., Sun P., Wang Y. (2021). Pear Defect Detection Method Based on ResNet and DCGAN. Information.

[66] Wu Q., Chen Y., Meng J. (2020). DCGAN-Based Data Augmentation for Tomato Leaf Disease Identification. IEEE Access.

[67] Hu G., Wu H., Zhang Y., Wan M. (2019). A low shot learning method for tea leaf’s disease identification. Comput. Electron. Agric..

[68] Ni J., Liu B., Li J., Gao J., Yang H., Han Z. (2022). Detection of Carrot Quality Using DCGAN and Deep Network with Squeeze-and-Excitation. Food Anal. Methods.

[69] Dewi C., Chen R.C., Liu Y.T., Yu H. (2021). Various Generative Adversarial Networks Model for Synthetic Prohibitory Sign Image Generation. Appl. Sci..

[70] Cai Q., Abdel-Aty M., Yuan J., Lee J., Wu Y. (2020). Real-time crash prediction on expressways using deep generative models. Transp. Res. Part Emerg. Technol..

[71] Mahmoud M.A.B., Guo P. (2019). A Novel Method for Traffic Sign Recognition Based on DCGAN and MLP With PILAE Algorithm. IEEE Access.

[72] Dewi C., Chen R.C., Liu Y.T., Jiang X., Hartomo K.D. (2021). Yolo V4 for Advanced Traffic Sign Recognition With Synthetic Training Data Generated by Various GAN. IEEE Access.

[73] Bajić F., Job J., Nenadić K. Chart Classification Using Simplified VGG Model. Proceedings of the 2019 International Conference on Systems, Signals and Image Processing (IWSSIP).

[74] Meyes R., Lu M., de Puiseau C.W., Meisen T. (2019). Ablation Studies in Artificial Neural Networks. arXiv.

[75] Lake B.M., Salakhutdinov R., Gross J., Tenenbaum J.B. (2011). One shot learning of simple visual concepts. Cogn. Sci..

[76] Marin I., Gotovac S., Russo M., Bozic-Stulic D. (2021). The Effect of Latent Space Dimension on the Quality of Synthesized Human Face Images. J. Commun. Softw. Syst..

[77] Heusel M., Ramsauer H., Unterthiner T., Nessler B., Hochreiter S. (2018). GANs Trained by a Two Time-Scale Update Rule Converge to a Local Nash Equilibrium. arXiv.

[78] Barratt S., Sharma R. (2018). A Note on the Inception Score. arXiv.

